# Evaluation of the vegetative properties of biopolymer- and plant fiber-solidified sandy soil

**DOI:** 10.1016/j.isci.2026.116921

**Published:** 2026-07-22

**Authors:** Dianzhi Feng, Dejiang Zhang, Jiaxu Jin, Bing Liang, Yong Wan

**Affiliations:** 1 School of Civil Engineering, Liaoning Technical University, Fuxin, Liaoning 123009, China; 2 School of Mechanics and Engineering, Liaoning Technical University, Fuxin, Liaoning 123000, China; 3 State Key Laboratory of Geomechanics and Geotechnical Engineering Safety, Institute of Rock and Soil Mechanics, Chinese Academy of Sciences, Wuhan, Hubei 430071, China

**Keywords:** Vegetative properties, Water retention, Biopolymer, Plant fiber, Sandy soil

## Abstract

A single 45-day indoor planting experiment was conducted to evaluate the vegetative properties of biopolymer- and plant fiber-solidified soil as an eco-friendly material, given that high biopolymer content may hinder plant growth. Using oat as the indicator species, the planting medium consisted of Yellow River silt stabilized with 1.5% xanthan gum and 0.6% jute fiber, prepared at three compaction levels. The study designed and proposed a layered planting structure comprising original residual soil, solidified soil, a grass-seedsowing layer, and a raw soil cover layer. Results showed that this structure achieved a germination rate above 85%, with moderate compaction promoting better growth. Soil temperature remained between 23°C and 28°C, matric suction was stable at approximately 15 kPa, and moisture content uniformly ranged from 10% to 20% across depths. This layered design mitigated surface hardening, enhanced hydrothermal transport, and improved water infiltration through root-formed channels, thereby enabling the functional application of solidified soil in ecological slope protection.

## Highlights

• A layered structure overcomes soil surface hardening in plant growth
• The layered structure maintains stable temperature and uniform moisture
• Root channels enhance permeability by four orders of magnitude

## Graphical abstract

**Figure:**
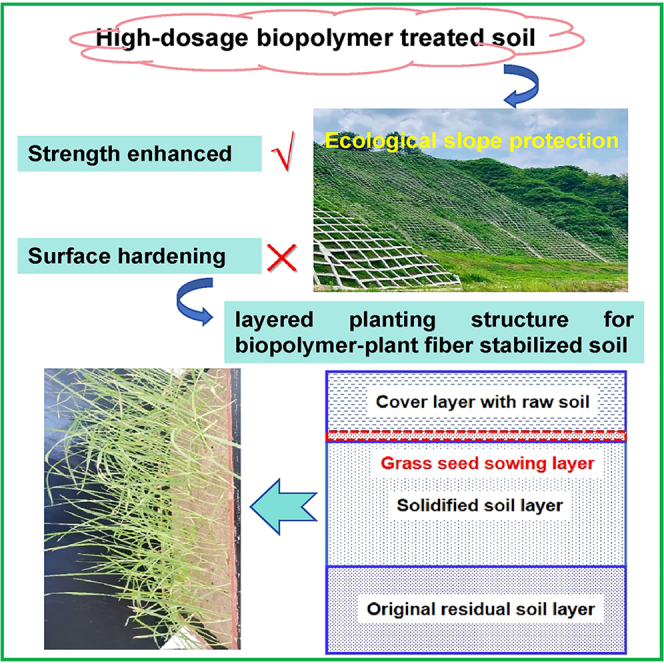

## Teaser

Soil science; Earth surface fluid properties; Plant biology

## Introduction

The Yellow River, known for having the highest sediment load in the world, generates a substantial amount of openly accumulated silt due to its perennial dredging projects. This accumulated silt not only occupies land but also readily triggers ecological and environmental safety issues, such as debris flows, dust emissions, and soil erosion.^1^^,^^2^ Therefore, achieving large-scale and high-quality ecological utilization of Yellow River silt as a resource has become an urgent engineering and environmental challenge. Against this backdrop, soil solidification technology has been extensively researched and applied. Conventional chemical cementitious materials, such as cement, are gradually being replaced by other technologies due to their high carbon emissions.^3^^,^^4^ Among the alternatives, the use of biopolymers combined with plant fibers to solidify Yellow River silt has emerged as a novel green geotechnical engineering technique.^5^^,^^6^^,^^7^^,^^8^ This approach establishes a new ecological pathway for engineering applications of solidified silt. It achieves this by enhancing the soil’s mechanical properties, improving its water retention, and creating a suitable growth matrix for plants.

Current research on the vegetative properties of solidified soil is predominantly focused on cement-based materials. For instance, the cement content in vegetation concrete is recommended to be controlled below 8% in order to balance strength and ecological requirements.^9^ However, the high alkalinity, high carbon emissions, and potential adverse effects of cement-based materials on soil ecosystems are increasingly debated, and their applicability in arid and water-scarce regions is also limited. Concurrently, global climate change has exacerbated drought and soil degradation. Many sand-fixation and desertification-control projects have struggled to achieve lasting effectiveness due to a failure to adequately address the complex technical challenges at the intersection of geotechnical engineering and ecology.

Given the extremely poor water retention and vast extent of sandy soil in this region, there is an urgent need, from the perspectives of engineering practicality and sustainability, to develop novel water-retaining and growth-promoting materials. All vital activities of plants depend on water. However, in sandy soil, water is easily lost through transpiration, guttation, and rapid infiltration, leading to water deficiency in the root zone, which becomes a key limiting factor for vegetation establishment.^10^^,^^11^^,^^12^ Biopolymer hydrogels, due to their strong hydrophilic nature, can significantly enhance the water-holding capacity of loose sandy soil when applied, thereby promoting plant growth.^13^^,^^14^ Furthermore, plant roots themselves can improve soil stability through their mechanical reinforcement effect.^15^ Therefore, developing solidified soil materials centered on biopolymers holds significant engineering application value for achieving water-saving ecological restoration on engineered slopes in arid regions.

In the context of application-oriented fundamental research for engineering, biopolymers and plant fibers have demonstrated considerable potential. Adding 0.5%–1.0% of dextran or xanthan gum (XG) can enhance the soil’s viscosity, improve the ability to resist surface erosion, and facilitate the improvement of soil’s water retention properties. This method can be regarded as a potential measure for desertification control projects in arid and semi-arid regions.^16^ Starch and XG hydrogels could serve as soil conditioners, improving the survival rate of engineered vegetation in arid environments.^17^ A new type of composite stabilizer developed based on carboxymethyl cellulose and XG not only enhanced the engineering properties of loess, such as compressive strength and water resistance, but also significantly promoted the growth of alfalfa.^18^ Another study shows that when slag soil is treated with a mixture adhesive containing 6% milk protein and 2% β-glucan, the corn plants grown thereon show significant improvements in terms of plant height, fresh weight, chlorophyll content, and enzyme activity.^19^ Hydrophilic Polysaccharide Biopolymer (HPB) was used to improve sandy soil. This action not only enhanced the soil’s erosion resistance but also provided a growth environment for herbaceous plants, thereby forming a biological polymer-plant integrated system suitable for slope stability.^20^ A newly developed XG-cellulose hydrogel has increased the soil’s water absorption capacity by approximately 60%, effectively reducing water evaporation, and the use of this improved topsoil cover helps promote plant growth.^21^ Regarding the engineering application of plant fibers, an appropriate amount of wood fiber was beneficial for the growth of herbaceous plants and could increase the shear strength of the root-soil composite, with a maximum enhancement of 47.20%.^22^ Biopolymers are also used to reinforce flood embankments. Even at very low concentrations of biopolymers, they can enhance soil strength and effectively protect the slopes of the embankments. This method not only improves the surface runoff capacity but also helps promote vegetation growth.^23^ Studies have shown that adding straw can promote seed germination and plant growth and increase vegetation coverage. Moreover, the preservation method of soaking straw in glue has no adverse effect on seed germination and growth.^24^

However, in engineering applications, if the type or dosage of the biopolymer is inappropriate, it can easily form a dense, hardened layer on the soil surface, which in turn hinders seedling emergence. Studies have shown that when different proportions of polymers were mixed into dry desert sand and cured for periods ranging from 3–90 days, the unconfined compressive strength (UCS) of most samples exceeded 1.0 MPa.^25^ The biopolymer solution tended to cement the surface layer of sand into a crust. The hardness and thickness of this crust depended on the material and its concentration. While beneficial for water retention, this crust could potentially affect seed germination. A low dosage of XG (≤0.5%) could enhance soil shear strength while promoting oat growth, whereas a high dosage resulted in hard cementation that impeded plant development.^26^ Another research also confirmed that excessive sand-fixing agents could cause surface hardening, affecting the emergence of wheat seedlings.^27^ Therefore, addressing the issue of surface over-congelation potentially caused by high dosages of biopolymers and finding the optimal balance between mechanical and ecological performance are crucial for exploring the practical application of high-dosage biopolymer-solidified soil in engineering revegetation.

Large-scale coal mining in northern China has created a vast number of exposed slopes and dump sites. These engineering-disturbed surfaces are ecologically fragile and unstable, thereby urgently requiring effective ecological restoration and slope protection technologies. This situation provides a clear and extensive application scenario for the resource utilization of solidified Yellow River silt. The core innovation of this research lies in designing the solidified sediment as an ecological planting substrate with the function of supporting plant growth, rather than merely using it as a filler or structural material. This directly meets the key engineering requirements for ecological restoration in complex terrains of arid and semi-arid regions. This substrate not only performs the traditional mechanical function of slope protection but also, through the synergistic action of biopolymers and plant fibers, creates a porous structure and an improved soil environment that provides ideal conditions for plant growth. Plant roots can penetrate deep into the substrate and the slope, forming a root-soil composite reinforcement system that significantly enhances the long-term stability and ecological integrity of the slope.^28^ This technological system combines environmental friendliness, vegetation-promoting properties, and excellent erosion resistance. During rainfall, the slope vegetation effectively slows surface runoff and promotes infiltration, thereby mitigating soil erosion and achieving an organic integration of engineering protection and ecological restoration. This study conducted indoor planter box planting experiments to simulate regional environmental conditions. Using oat as an indicator plant, the study systematically monitored growth indicators such as germination rate and plant height. Combined with the dynamic changes of internal soil temperature, moisture content, and matric suction, the study comprehensively evaluated the vegetative properties of Yellow River silt co-solidified with biopolymers and plant fibers. The purpose of this study is to provide direct scientific evidence and technical support for the resource utilization of Yellow River silt and the ecological protection of engineered slopes in arid regions.

## Results

### Planting pre-experiment results

**Figure 1:**
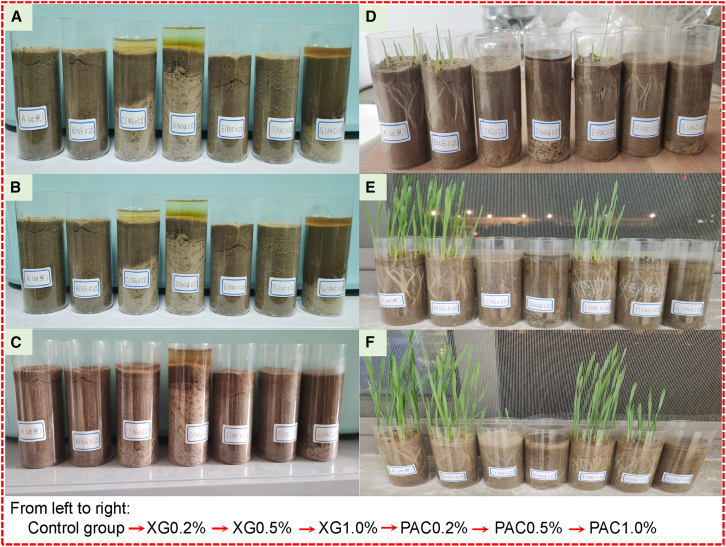
Water infiltration in soils treated with different biopolymers dosages (A) 24 h, (B) 48 h, (C) 72 h, (D) 96 h, (E) 144 h, and (F) 192 h.

The experimental results showed that after standing for 72 h, water in the samples treated with XG at dosages exceeding 0.2% had still not completely infiltrated, as shown in Figure 1. For samples with a biopolymer dosage of 1.0%, even after accounting for potential water evaporation, standing water that had not infiltrated remained in the plastic cups. In the control group, the complete infiltration time for 30 mL of water was only about 5 s, which is consistent with the results of the disintegration tests previously conducted by the research team.^5^ After 192 h of observation, the germination rate in both the 0.2% biopolymer-treated group and the control group was relatively high, at approximately 90%, and plant growth was also comparatively better. However, no seed germination was observed in samples with XG and polyanionic cellulose (PAC) dosages of 0.5% and 1.0%. The primary reason is that the reduced permeability of the soil treated with high dosages of biopolymer severely inhibited seed germination and growth due to insufficient soil aeration. As moisture was lost from the planting substrate, the soil gradually hardened from top to bottom, exhibiting noticeable surface crusting.

Therefore, in the practical engineering application of biopolymer-solidified soil, meeting strength requirements necessitates increasing the biopolymer dosage. Simultaneously, to serve effectively as a planting substrate, two key issues must be considered. The first is surface layer hardening, which severely inhibits seed germination. The second is the difficulty of water infiltrating and supplying moisture to the deeper soil for plant growth after germination.

### Proposal of layered planting structure

**Figure 2:**
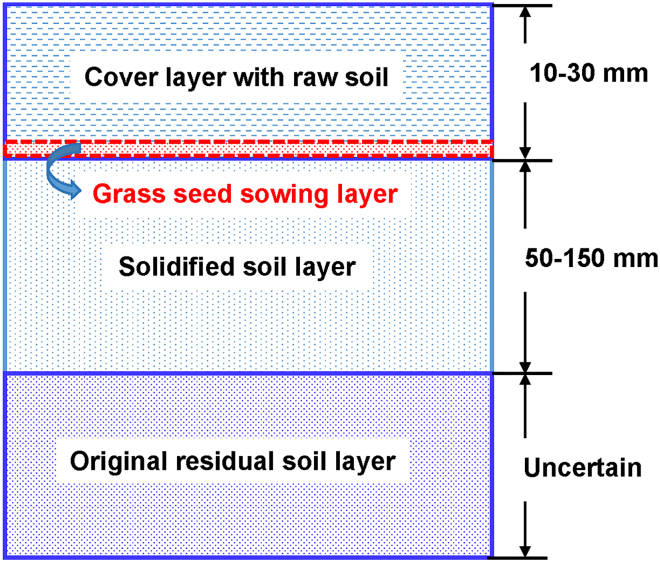
Design of layered planting structure using biopolymer-based solidified soil

After multiple experimental trials and literature reviews, the research team proposes a novel layered planting structure design for high-dosage biopolymer-based solidified soil, as illustrated in Figure 2. The designed structure consists of four distinct layers arranged from bottom to top: the original residual soil layer, the solidified soil layer, the grass seed sowing layer, and the cover layer with raw soil. Its key features are as follows.

(1) The original residual soil layer is the native soil or slope material exposed in the natural environment. The thickness of soil available for plant growth within this layer is typically a specific value, which varies according to different practical engineering conditions.
(2) The solidified soil layer is formed by treating the native soil with materials such as biopolymer. The designed thickness for this layer is approximately 50–150 mm. This thickness is adjusted based on the slope gradient. If the layer is too thin, the water retention capacity of the treated soil is poor; if it is too thick, it can lead to a significant increase in treatment costs.
(3) The grass seed sowing layer is placed directly above the solidified soil layer, and its thickness is negligible. It is worth noting that the appropriate grass seed should be selected based on the specific engineering application scenario, and it should also be treated, such as being soaked for a certain period of time.
(4) The design of the cover layer with raw soil is a key focus of this planting structure. Previous methods often used solidified native soil for this top layer as well, leading to issues such as surface hardening and poor water infiltration. By designing it as a cover layer with raw soil, externally supplied water can promptly infiltrate into the soil body. The surface layer of soil, treated with biopolymer and plant fibers (and serving as the seeding zone), maintains adequate moisture under arid or semi-arid conditions, thereby resisting hardening and clump formation. Meanwhile, the seeds, positioned within this layer, can readily absorb external water supply. After germination, the roots can grow deeper into the solidified soil layer to access water and nutrients. Furthermore, from the perspective of resource utilization of the soil to be treated, this design also helps increase the utilization rate of the vast amounts of dredged sediment stockpiled in nature. The cover layer with raw soil can be designed with a thickness of 10–30 mm. This thickness should be adjusted according to various parameters, such as the natural slope gradient, the internal friction angle of the soil material, and the type of plants to be cultivated. If the layer is too thin, seeds may be directly exposed to the environment during actual implementation. This not only wastes seeds but also makes them vulnerable to being spotted and consumed by birds, and it can drastically reduce the survival rate of germinated plants due to conditions like wind erosion, thereby compromising the ecological revegetation effect on the slope. Conversely, if the cover layer with raw soil is designed to be too thick, it can lead to dust emissions under wind erosion and cause severe surface runoff during water supply, while also hindering the normal growth of plants after seed germination.

### Application of layered planting structure

For the planting structure layer designed for biopolymer-based solidified soil as described earlier, the corresponding planting method can be summarized into the following four operational steps.

Step 1: based on practical engineering requirements, remove large debris, such as bulky gravel, from the original residual soil layer. Subsequently, spray water onto the surface of the original residual soil layer. The purpose is to replenish initial moisture of this layer and to increase the friction and workability for the subsequent placement of the solidified soil layer.
Step 2: after mixing the biopolymer or plant-fiber materials with the native soil at a specified water content, construct the solidified soil layer according to the designated placement thickness and compaction parameters.
Step 3: create several staggered and regular grooves on the surface of the solidified soil layer by roughening. This facilitates the even distribution of seeds during sowing and prevents seeds from rolling down the soil slope, which could affect the revegetation outcome. Finally, sow the plant seeds at the specified density.
Step 4: spread the calculated mass of native soil onto the cover layer with raw soil, based on parameters such as the cover soil thickness. Moisten the cover layer with raw soil using spray irrigation. Before seed germination and emergence, appropriate supplemental watering is necessary to maintain sufficient moisture between the cover layer with raw soil and the solidified soil layer, thereby preventing hardening. After emergence and growth begin, the intervals for subsequent watering to support plant growth can be extended.

After plant emergence and growth begin, even under prolonged drought conditions in the natural environment, the hardening of the cover layer with raw soil and the surface of the solidified soil layer can effectively lock in moisture within the soil mass, preventing significant water loss. The moisture retained within the solidified soil layer is then available for root uptake and plant growth. At this stage, the water retention performance of the biopolymer-based solidified soil material is fully demonstrated. Furthermore, when precipitation occurs naturally or water is supplied through artificial irrigation, the external moisture is stored by the cover layer with raw soil. This increases the residence time of water on the solidified soil layer, allowing more water to infiltrate along the plant stems into the deeper solidified soil layer, thereby fulfilling its water conservation function.

### Indoor model box planting experiment

**Figure 3:**
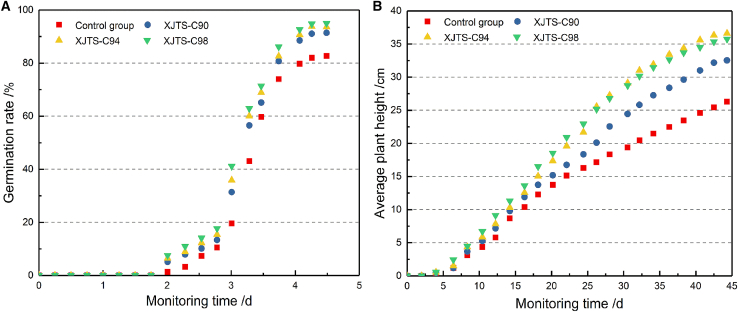
Variation in germination and average plant height in the indoor planting test (A) The variation in germination rate. (B) The variation in the average plant height.

Based on the experimental records, the germination rate (recorded as the number of seedlings that broke through the surface of the cover layer with raw soil) and the average plant height of oat seeds during the indoor planting test were plotted, as shown in Figures 3A and 3B, respectively.

Under the four different planting conditions, the germination rate stabilized approximately before the 4^th^ day after sowing. The control group exhibited a germination rate of around 82%, whereas the Yellow River silt solidified with XG and jute fiber (XJTS) substrates with compaction levels of 90%, 94%, and 98% achieved germination rates of 90%, 92%, and 95%, respectively. The control group exhibited a generally slower germination rate overall, resulting in a relatively lower final germination rate. Notably, no water was supplemented during the approximately 5-day period when the germination rate was monitored after sowing, and there were no significant differences among the selected oat seeds used for sowing. The primary reason is attributed to the rapid evaporation and dissipation of water in the control group, which limited the availability of sufficient moisture for the oat seeds during water absorption and germination.^25^ In contrast, the inherent moisture within the XJTS substrates demonstrated a strong self-retention capacity. Furthermore, during evaporation, the oat seeds could continuously access moisture from the underlying solidified soil layer, thereby meeting their requirements for germination and sprouting.

As shown in Figure 3B, over the monitoring period of approximately 45 days, the trends in average plant height for oats grown in the four different substrates followed a similar pattern: an initial rapid increase followed by a gradual deceleration in the growth rate. Compared to the planting boxes containing XJTS substrates with different compaction levels, the average plant height increase in the control group began to slow down around the 20^th^ day. The direct cause of this situation lies in the fact that the soil in the control group lacks biopolymers and plant fibers, and the cohesion between soil particles is weak, making it difficult to provide strong support for the plant roots. The structure formed by the encapsulation of soil particles by biopolymers and plant fibers is essential for ensuring the stability of plants on the ground.

**Figure 4:**
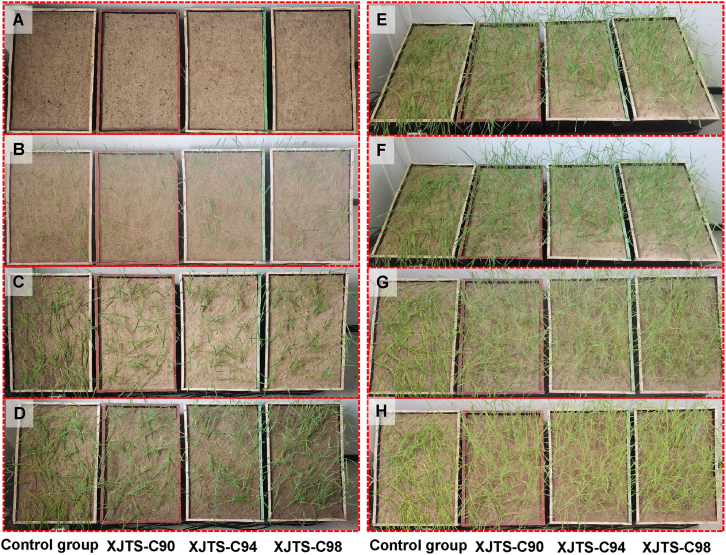
Growth of oats at different times (A) 5 days, (B) 10 days, (C) 15 days, (D) 20 days, (E) 25 days, (F) 30 days, (G) 35 days, and (H) 45 days.

Figure 4 shows the growth status of oats at different time points. Lodging occurred earlier in the control group (as shown in Figure 4D). After lodging, further plant growth was significantly inhibited. During the experimental observation period, the average plant height of oats in the control group was 26.2 cm, while for the XJTS substrates with compaction levels of 90%, 94%, and 98%, the average plant heights reached 32.5, 36.0, and 35.5 cm, respectively. The degree of compaction also influenced the variation in average plant height. When the compaction level was too high, the downward growth of plant roots was hindered. During subsequent water supply, it was difficult for the roots to promptly absorb and store a higher proportion of the externally supplied infiltrating water into the solidified soil layer. Conversely, when the compaction level was too low, the rate of water evaporation and dissipation increased relatively. The bonding capacity between the developed root system and the soil substrate was also weakened, even leading to a tendency for lodging during growth.

The initial water content of the planting substrate in the solidified soil layer was controlled at 17%. For subsequent water supplementation, the established experimental protocol was strictly followed: each planting box received 1000 mL of water via a spray bottle at the time of sowing. After the germination rate stabilized (around day 4), the second water supplementation was initiated, with 500 mL sprayed into each box. Thereafter, 500 mL of water was added to each box every 5 days. Each water supplementation event was scheduled after 8:00 p.m., with the spraying duration for each box controlled to approximately 10 min. By the end of the indoor planting experiment, the total amount of externally supplied water per box was 5,500 mL.

**Figure 5:**
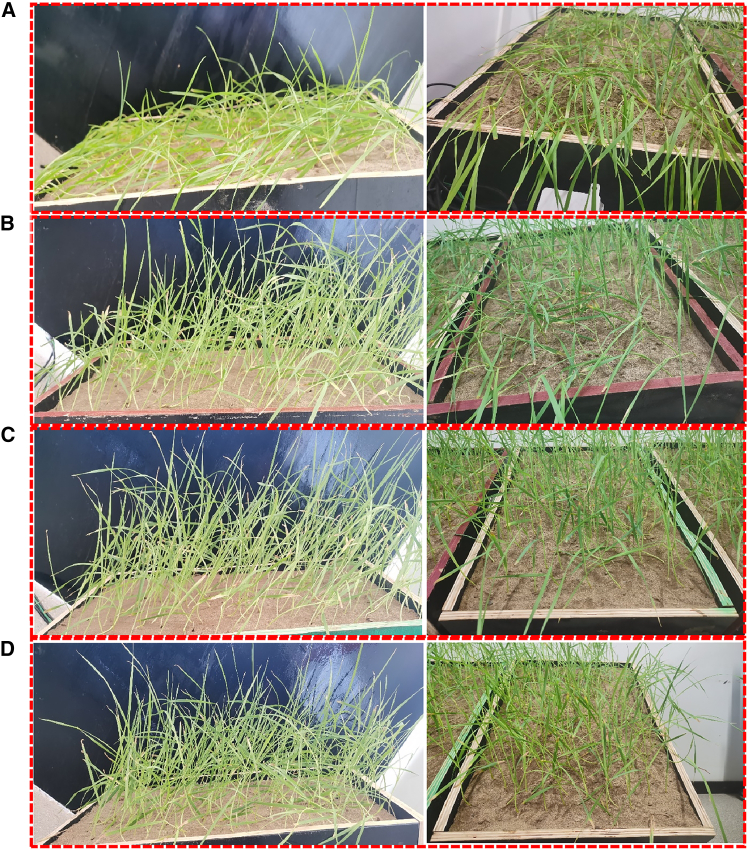
Health status of oats after 45 days of growth and soil erosion on surfaces of different soil substrates (side view) (A) Control group; (B) XTJS-90, (C) XTJS-94, and (D) XTJS-98.

As shown in Figure 5, the health status of oats after 45 days of growth and the soil erosion on the surfaces of different soil substrates are presented from a side-view angle. It should be noted that, to focus on studying the effect of different planting substrates on plant growth, no fertilizers were added to any of the substrates in the indoor planting boxes. Consequently, yellowing of the oat plants was observed during growth. Water supplementation during the planting process had certain effects on the soil surfaces of the different substrate types. For example, on the surface of the control group (Figure 5A), the growing plants could stabilize a portion of the dredged sediment (a sandy silt). However, the external spraying process caused a certain amount of sandy soil to slide downward. The combined result was the formation of pitted depressions on the soil surface in the control group. In substrates with higher compaction levels, the phenomenon of sandy soil sliding and accumulating at the bottom of the planting boxes was also observed, as shown in Figure 5D. This can be explained by the excessively high compaction: during water supplementation, after passing through the cover layer with raw soil, water reached the surface of the solidified soil layer. The proportion of this water that could promptly infiltrate into the interior of this layer was relatively low. Consequently, excess water carried fine soil particles to slide down the slope surface. No similar phenomenon was observed in the case of lower compaction levels, where the soil surface remained relatively even.

## Discussion

### Characteristics of soil temperature variation during plant growth

**Figure 6:**
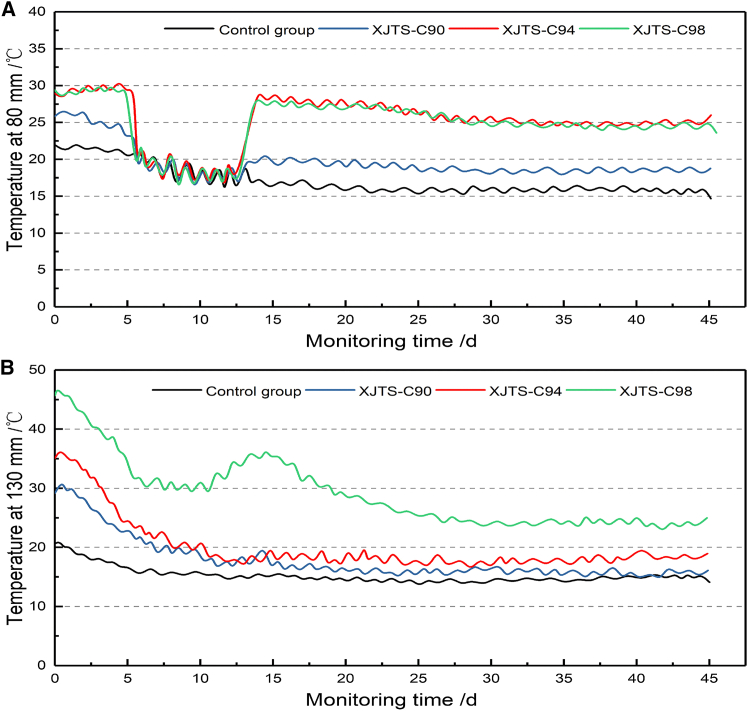
Changes in soil temperature at different locations during plant growth (A) Variations in soil temperature at 80 mm depth. (B) Variations in soil temperature at 130 mm depth.

After processing the collected data, the variations in soil temperature at different positions (80 and 130 mm heights from the bottom of the designed planting structure layer) during plant growth are plotted in Figure 6. Overall, the four different planting substrates exhibited similar variation patterns at the different measurement positions.

As shown in Figure 6A, the internal soil temperature at the 80 mm position remained at a stable level for the first 5 days, showed a general declining trend after day 5, and then stabilized within a constant range after approximately day 13. This phenomenon is likely attributed to the gradual development and maturation of the plant root system between days 5 and 13. The oat roots, distributed throughout the planting substrate, created pathways connecting the substrate interior to the external environment, potentially leading to the maintenance of a lower temperature.^29^ After day 13, as moisture within the substrate was consumed, the interwoven network of plant roots present in the soil played a role. In substrates with higher compaction levels, such as XJTS-C94 and XJTS-C98, the plant fibers and root systems provided better insulation, maintaining the internal soil temperature between 23°C and 28°C. In contrast, for the control group that contained no plant fibers, the soil temperature remained around 15°C after day 13. During the experiment, oat plants in all four boxes were exposed to daily sunlight, with slight variations in exposure duration. This resulted in noticeable diurnal fluctuations in the daily monitoring data. The 130 mm position was relatively closer to the substrate surface, and consequently, soil temperatures at this location were generally higher.

After the initial 5-day growth period, the internal temperatures of the four different substrates—control group, XJTS-C90, XJTS-C94, and XJTS-C98—decreased from their initial values of 47.5°C, 35.1°C, 29.5°C, and 21.3°C–25.3°C, 19.8°C, 16.3°C, and 13.2°C, respectively. For the substrate with the highest compaction level (XJTS-C98), the soil temperature at the 130 mm position remained at a relatively higher level during the later stages of plant growth.

### Characteristics of moisture content variation during plant growth

**Figure 7:**
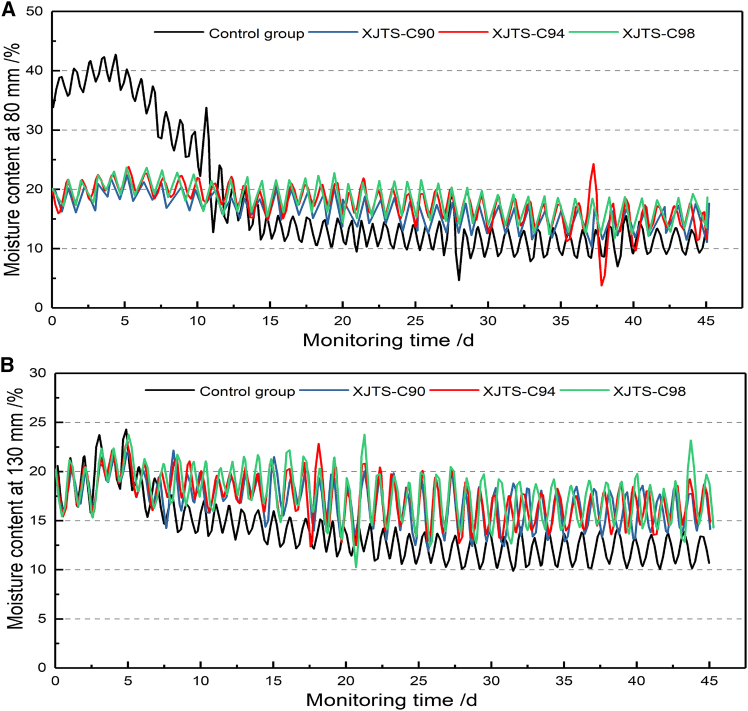
Changes in soil moisture content at different locations during plant growth (A) Variations in moisture content at 80 mm depth. (B) Variations in moisture content at 130 mm depth.

Generally, the enhancement effect of biopolymers on soil moisture content originates from their hydrophilic functional groups (e.g., OH^−^), which increase the soil’s hydrophilicity and improve its water-retention capacity.^30^ The variations in soil moisture content at different positions during plant growth are shown in Figure 7. It can be observed that during the first 5 days of plant growth, the internal soil moisture content at the 80 mm position (i.e., the interface between the solidified soil layer and the cover layer with raw soil) showed a continuous increasing trend. This trend was particularly pronounced in the control group, where the moisture content increased from 34.2% to 43.5%, representing a 27.19% increase, as shown in Figure 7A. This is attributed to the gradual downward migration and accumulation of initial moisture within the planting substrate during this period, leading to the increasing trend at this location. Subsequently, the moisture content gradually decreased due to water consumption by the growing oats and losses from evaporation. However, the periodic water supply in the experimental design and the regular daily natural light conditions caused the subsequent moisture content at different positions to fluctuate sinusoidally within a range over time.

The trend of internal soil moisture content variation at the 130 mm position (i.e., within the solidified soil layer) was similar to that at the 80 mm position, as shown in Figure 7B. However, the change in moisture content for the control group during the first 5 days was relatively more gradual. The design of the cover layer with raw soil helped maintain the soil moisture content at the surface of the solidified soil layer within a range of 15%–20% during plant growth. This effectively prevented the surface hardening of the biopolymer-based solidified soil and its adverse effects on plant growth.

Furthermore, under natural conditions, the moisture content in the lower soil layer is typically higher than that in the upper layer. In this experiment, however, when using biopolymer- and plant fiber-solidified dredged sediment as the planting substrate, the internal soil moisture content at different positions showed minimal disparity and no significant fluctuations. This is mainly due to the combined moderating effect of the cover layer with raw soil and the plants themselves on the internal soil moisture, which promoted a maximally uniform distribution of moisture within the planting substrate. The results from the moisture content variations confirm the practical utility of the designed cover layer with raw soil within the planting structure layer.

### Characteristics of matric suction variation during plant growth

Matric suction arises in unsaturated soils due to the simultaneous presence of water and air within the pores. Surface tension exists at the air-water interface (the contractile skin), causing the pore air pressure to differ from the pore water pressure. Typically, the pore air pressure is greater than the pore water pressure, resulting in matric suction.^31^ Matric suction is a key indicator of the water absorption capacity of unsaturated soil and is closely related to soil moisture content. Generally, a lower moisture content corresponds to a higher matric suction. Matric suction has important applications in soil science and geotechnical engineering, influencing plant growth, land use, and the mechanical properties of unsaturated soils.^32^

**Figure 8:**
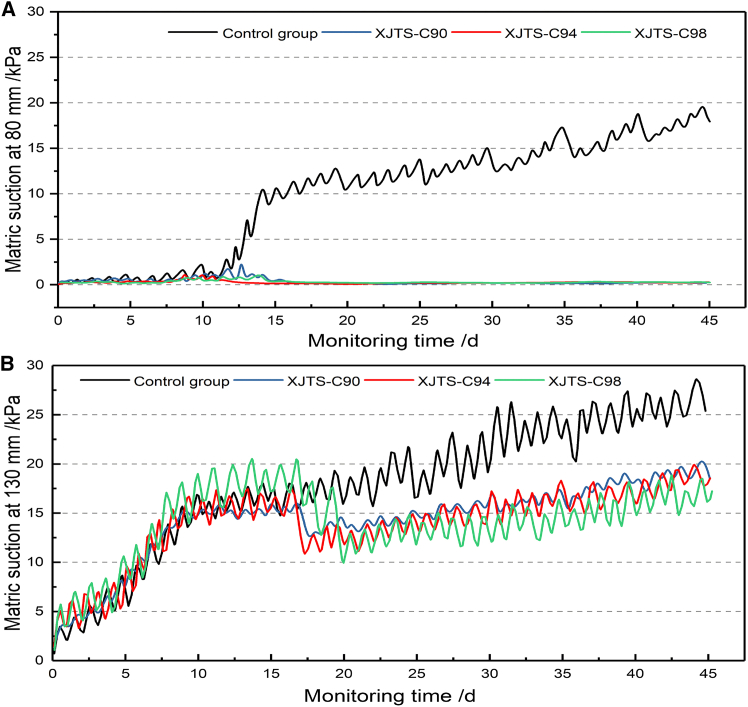
Changes in matric suction at different locations during plant growth (A) Variations in matric suction at 80 mm depth. (B) Variations in matric suction at 130 mm depth.

Figure 8 presents the variations in matric suction at different locations during plant growth. It can be observed that at the 80 mm position, the matric suction in the control group did not change significantly before the 11^th^ day, remaining stable within a range of 0–2 kPa. Subsequently, due to the accelerated consumption of internal moisture caused by the extension and distribution of plant roots within the soil, the matric suction increased rapidly. During the subsequent plant growth period, the matric suction in the control group maintained a relatively gradual increasing trend overall, reaching approximately 20 kPa after 45 days. In contrast, for the other three biopolymer-based solidified soil substrates with different compaction levels, the matric suction at this position showed only minor variations. Owing to the infiltration of externally supplied water and their inherent water retention capacity, the matric suction remained at zero for an extended period.

At the 130 mm position, during the initial stage of plant germination and growth, the matric suction for all four different planting substrates increased steadily within the first 10 days, rising from 0 to approximately 15 kPa. Subsequently, from around day 10 to day 16, it fluctuated within a stable range. Following the full development of the plant root system and the establishment of connections between the plant stems and the external environment of the substrate, the infiltration pattern of externally supplied water changed significantly. As water continuously entered the soil, the matric suction of the biopolymer-based solidified soil substrates began to decrease. Taking the XJTS-C98 substrate as an example, its matric suction decreased from a maximum of 20.3 kPa to around 10.1 kPa. After day 17, the matric suction for all substrates showed a trend of continuous increase. However, compared to the control group, the biopolymer-based solidified soil substrates exhibited smaller-magnitude changes and no significant numerical fluctuations overall.

### Variations in permeability coefficient

To further verify the efficacy of the designed planting structure layer in addressing the surface hardening issue of biopolymer-based solidified soil, soil sampling was conducted after the completion of the indoor planting test in model boxes. A cutting ring was used to collect samples from the vegetated soil to investigate changes in the soil permeability coefficient before and after plant growth. During sampling, the oat plants above the soil surface were carefully trimmed off without disturbing the soil structure. A soil sampler was then employed to press the cutting ring (61.8 mm in diameter and 40 mm in height) into the planting substrate. It is important to note that each soil specimen for permeability testing was ensured to contain the same number of oat plants, and these plants were in a similar growth stage. This is crucial because different growth stages correspond to variations in root and stem development, which can significantly affect the accuracy of permeability measurements. The calculated permeability coefficients before and after planting are presented in Table 1.

**Table 1:** Variation of permeability before and after the plant test

Proportion of planting substrate	Before planting (cm/s)	After planting (cm/s)
Control group	1.37 × 10^−3^	2.38 × 10^−2^
XJTS-C90	1.17 × 10^−5^	6.56 × 10^−3^
XJTS-C94	1.08 × 10^−6^	3.62 × 10^−3^
XJTS-C98	9.11 × 10^−7^	1.41 × 10^−3^

As shown in Table 1, before the commencement of the indoor planting experiment, the permeability coefficient of the raw soil was 1.37 × 10^−3^ cm/s. As the compaction level increased from 90% to 98%, the permeability coefficient decreased from 1.17 × 10^−5^ to 9.11 × 10^−7^ cm/s. This indicates that different compaction levels can significantly influence the permeability coefficient. However, after the planting experiment concluded, the permeability coefficient of the raw soil increased by approximately one order of magnitude to 2.38× 10^−2^ cm/s. For the biopolymer- and plant fiber-solidified soils with different compaction levels, even more pronounced changes in the permeability of the substrate occurred after the planting experiment. Taking XJTS-C98 as an example, its permeability coefficient increased from 9.11 × 10^−7^ to 1.41 × 10^−3^ cm/s, representing an increase of nearly four orders of magnitude. This can be attributed to the connectivity provided by plant roots and stems, which created rapid pathways for external water to pass through the soil mass, thereby significantly altering its permeability. Generally, treating soil with a low dosage of biopolymer alone can effectively reduce its permeability.^33^ However, this study employed a high biopolymer dosage. It relies on the connectivity established by the root system to enhance water infiltration capacity, thereby enabling the ecological slope protection application of soil solidified with a high dosage of biopolymer and plant fibers.

The permeability test results from the indoor planting experiment indicate that externally supplied water can first be stored in the cover layer with raw soil. It then infiltrates into the solidified soil layer (i.e., the biopolymer-based solidified soil planting substrate) via the pathways created by plant roots and stems. Subsequently, the inherent water-retention capacity of the material within the solidified soil layer can provide further moisture for plant growth. These experimental results confirm the effectiveness of the designed planting structure layer, as it successfully addresses the problem of surface hardening in biopolymer-based solidified soil materials, which would otherwise hinder normal plant growth.

### Limitations of the study

This study is a short-term indoor model box planting experiment. It proposes a planting structural layer that uses soil solidified with a high dosage of biopolymer as the planting substrate. The experiments were conducted under controlled indoor conditions. Therefore, uncertainties remain when extrapolating the results to engineering applications. Our research team has conducted some preliminary experiments in practical engineering contexts. Although these results have not yet been reported, they initially confirm that the findings of this paper can be applied effectively. Future research should include field planting trials in arid and semi-arid regions. These trials can select suitable plant species according to local vegetation characteristics. Long-term monitoring data should be collected to record basic plant growth parameters. Soil nitrogen, phosphorus, and potassium contents should also be measured. Such data will help evaluate the water-saving performance of the planting structural layer. They will also help assess the durability of biopolymers in the soil. Ultimately, this work can provide a decision-making basis for ecological slope protection in mining areas.

## Resource availability

### Lead contact

Requests for further information and resources should be directed to and will be fulfilled by the lead contact, Wan Yong (coolfengdz@163.com).

### Materials availability

This study did not generate new unique reagents.

### Data and code availability

• The research data used in the paper are available in the Mendeley Data, with the connected network address listed in the key resources table.
• This paper does not report original code.
• Any additional information required to reanalyze the data reported in this paper is available from the lead contact upon request.

## Acknowledgments

This research was supported by the following grants: (1) The Major Science and Technology Program of Inner Mongolia, China (2021ZD0007); (2) The Basic Research Project of the Department of Education of Liaoning Province (LJ212510147017); and (3) Wuhan Special Zone Project of the Natural Science Foundation of Hubei Province, China (2024040701010061).

## Author contributions

F.D., conceptualization, methodology, formal analysis, writing – original draft, and funding acquisition; Z.D., writing – review and editing and investigation; J.J., visualization and writing-review; L.B., visualization; W.Y., project administration, supervision, and funding acquisition.

## Declaration of interests

The authors declare no competing interests.

## Declaration of generative AI and AI-assisted technologies in the writing process

During the process of writing this manuscript, the authors used DeepSeek for language polishing. After using this tool, the author conducted necessary reviews and revisions to the content and take full responsibility for the content of the publication.

## STAR★Methods

### Key resources table

**Table:** 

REAGENT or RESOURCE	SOURCE	IDENTIFIER
**Biological samples**		

Yellow River silt	Wuhai City, Inner Mongolia Autonomous Region	N/A
Oat seeds	Bai City Academy of Agricultural Sciences	Baiyan 7
Xanthan gum	Shanghai Maclin Biochemical Technology Co., Ltd	g810381
Polyanionic cellulose	Shanghai Maclin Biochemical Technology Co., Ltd	P875778
Jute fiber	https://detail.1688.com/	N/A

**Software and algorithms**		

Origin	Version of 2017	https://www.originlab.com/

**Other**		

Sensors for temperature and moisture content data	Shandong Renke Technology Co., Ltd	https://rkckth.cnreagent.com/
Matric suction sensor	Hainan Lingyun Technology Co., Ltd	http://www.ascod.cn/
Indoor growing conditions (25°C, a relative humidity of 75%)	N/A	N/A
Research data	Mendeley Data	https://data.mendeley.com/drafts/x37mkpk8d6

### Experimental model and study

#### Research area

The specific engineering area focused on this study is located in Wuhai City, Inner Mongolia Autonomous Region, with geographical coordinates between approximately 106.36°–107.05°E longitude and 39.15°–39.52°N latitude. Figure S1 presents data on temperature and precipitation variations over the past five years, highlighting the region’s prominent characteristics of significant temperature fluctuations and scarce rainfall.^34^ The Yellow River flows through the urban area of Wuhai City, forming Wuhai Lake. The annual dredging of this lake generates a massive amount of silt. Furthermore, intensive open-pit coal mining activities around Wuhai City have resulted in numerous exposed slopes requiring treatment. Due to the arid and rain-scarce climate in this region, natural vegetation recovery is difficult, and the effectiveness of conventional greening projects is severely limited. Following the solidification treatment of Yellow River silt, and adhering to the principle of adapting measures to local conditions, the vegetative properties become one of the key indicators for evaluating whether such new solidified soil materials can be successfully applied in local ecological slope protection projects.

#### The Yellow River silt

The Yellow River silt was collected from the research area, with an initial water content of 5.32% and a maximum dry density of 1.67 g/cm^3^. Most soil particles range from 0.005 to 0.075 mm in size, and approximately 5% of the clay particles is contained. According to the Unified Soil Classification System, the soil can be classified as the silty sand. More detailed data can be found in the previously published research results.^7^

#### Biopolymers

XG is a microbial biopolymer produced by Xanthomonas campestris. The XG used in the experiment, with an ash in 5.5–16.0% and a drying loss less than 15%, presents white to light yellow powder, and its molecular formula is C_35_H_49_O_29_. The polyanionic cellulose (PAC) used in the pre-planting experiment is a water-soluble, anionic cellulose ether derived from natural cellulose. Its molecular formula is generally represented as [C_6_H_7_O_2_(OH)_2_CH_2_COONa)]n. Both the XG and PAC are produced by Shanghai Maclin Biochemical Technology Co., Ltd.

#### Jute fibers

The plant fibers used in the experiment were jute fibers (JF), and the length of the jute fibers was 20 mm. The trimmed JF was immersed in 5 wt % sodium hydroxide solution for 6 h, and then thoroughly washed with deionized water, finally dried in an oven at 50°C and sealed for further use. More detailed data can also be found in the previously published research results.^7^

### Method details

#### Planting pre-experiment

To assess the suitability of using biopolymer-treated Yellow River silt as a planting substrate, a preliminary micro-scale indoor planting experiment was conducted in transparent cylindrical plastic cups (40 mm in diameter, 100 mm in height). The laboratory conditions were maintained at approximately 25°C and 75% relative humidity. The specific experimental procedure was as follows: two typical biopolymers, XG and PAC, were each mixed with the Yellow River silt at dosage rates of 0.2%, 0.5%, and 1.0% by mass of the dry silt. The treated soil mixtures were then placed into the plastic cups, ensuring a consistent fill height in each. An equal quantity of oat seeds was sown onto the surface of the soil in each cup. Subsequently, the seeds were covered with a 1 cm thick layer of sand treated with the corresponding biopolymer at the original dosage. To provide a clearer contrast for observing plant growth variations, a separate control group was established using untreated silt. Finally, to minimize surface disturbance from watering, 30 mL of tap water was applied uniformly using a small spray bottle. During the subsequent observation period, no additional water was supplied to any experimental group, and the water infiltration and plant growth were monitored continuously. When filling the planting substrate in the transparent cylindrical plastic cups, a thin layer of vaseline was applied inside the cylinder to reduce the influence of the contact surface between the cylinder side wall and the soil on the determination of permeability.

#### Indoor planting test in model boxes

Based on previous research findings, the planting soil for the indoor planting test in model boxes consisted of Yellow River silt solidified with XG and jute fiber (XJTS). The optimal mix proportion identified in prior studies was selected, with an XG dosage of 1.5% (by mass of dry sand), a jute fiber dosage of 0.6% (by mass of dry sand), and a jute fiber length of 20 mm.^7^ The maximum dry density for this optimal XJTS mix was determined to be 1.66 g/cm^3^, with an optimum water content of 17%. Choosing the appropriate compaction degree is beneficial for enhancing the strength in practical applications and also promoting plant growth. For the indoor planting test in model boxes, a total of four observation boxes were designed. Corresponding to earlier research results, these were set as the control group, XJTS-C90, XJTS-C94, and XJTS-C98, representing three different levels of compaction.^6^

A flowchart of the indoor planting test using model boxes is presented in Figure S2. First, as shown in Figure S2A, wooden model boxes were custom-made with dimensions of 600 cm in length, 400 cm in width, and 200 cm in height. Geogrids were fixed to the bottom and both inner sidewalls of the boxes to increase friction between the box interior and the XJTS material. Markings were made on the sidewalls at heights of 80 mm, 130 mm, and 150 mm from the bottom. Openings, approximately 40 mm by 25 mm, were created on one side at the 80 mm and 130 mm levels to facilitate subsequent sensor cable routing.

Next, as illustrated in Figure S2B, the XJTS material was prepared by uniformly mixing air-dried and sieved Yellow River sediment, sodium hydroxide-pretreated and dried jute fibers, and XG, with its water content controlled at 17%. The mixture was then placed into the planting model boxes. It is noteworthy that no additional nutrients were added to the XJTS material in this planting test to minimize experimental observation errors. Using the markings at 80 mm, 130 mm, and 150 mm as guides, the calculated mass of the moist XJTS mixture was placed and compacted in layers.

The first layer of sensors was embedded at the 80 mm marking, a location within the solidified soil layer. At the 130 mm marking, which corresponds to the grass seed sowing layer adjacent to the solidified soil layer, the second layer of sensors was embedded. Following this, regular grooves were created on the soil surface, and oat seeds were sown immediately with a spacing of approximately 40 mm between adjacent seeds, as shown in Figures S2C and S2D.

Oat was selected to evaluate the suitability of XJTS as a planting substrate due to its strong climate adaptability, high drought resistance, well-developed root system, rapid growth, simple cultivation technique, and relatively low cost.^35^^,^^36^ To better simulate field conditions, the oat seeds used in the experiment were not pre-germinated. After sowing the seeds, a 20 mm thick layer of dredged sediment was added to form the cover layer with raw soil and leveled with a spatula. Approximately 1000 mL of water was then initially sprayed onto the cover layer with raw soil in each model box using a watering can. Finally, the four planting boxes were slowly tilted and secured at a specified position, forming a 60° slope angle with the horizontal ground, as shown in Figure S2G. This was achieved by marking and aligning reference lines between each box, the horizontal ground, and a vertical wall. Control the indoor environmental conditions, maintaining the temperature at approximately 20–25°C and the humidity at 30%–55%. Natural light sources were selected, and the daily lighting duration was approximately 9 h on sunny days.

A diagram illustrating the sensor layout within a single planting box for the indoor planting test in model boxes is presented in Figure S3. Temperature and moisture content data were measured using a combined probe-type sensor capable of simultaneously monitoring temperature, moisture, pH, and electrical conductivity. The sensors and the corresponding data loggers were purchased from Shandong Renke Technology Co., Ltd. The main focus of this part is to monitor the changes in the internal temperature and moisture content of the soil. The sensor specifications include a temperature resolution of 0.1°C and a moisture content resolution of 0.01. For measuring matric suction, WATERMARK 200SS porous matrix soil moisture sensors were employed, offering a measurement range of 0–199 kPa and a resolution of 0.1 kPa. These sensors consist of corrosion-resistant electrodes embedded within a granular matrix, and they measure soil matric suction via a solid-state electrical resistance sensing device. The data loggers for the matric suction sensors were provided by Hainan Lingyun Technology Co., Ltd.

The first layer of sensors was embedded at the 80 mm mark, and the second layer was embedded at the 130 mm mark. Within each layer, the two sensors were spaced 50 mm apart. Prior to installing the sensors in the four model boxes, the wiring was organized and labeled, and the data loggers were configured and calibrated, as shown in Figure S2F. Once all sensors and data loggers were in place, power was supplied uniformly to the system, and experimental data recording commenced.

### Quantification and statistical analysis

Parameters such as the germination and growth of oat seeds by using the five-point sampling method. For example, when measuring the average height of the plants, five oat plants were selected from top to bottom in each planting box and marked. Then, the average value of these plants was taken as the data for the average plant height.

During the subsequent plant growth process, changes in soil matrix suction, soil temperature, and moisture content were all monitored and recorded. From the large amount of data obtained by the sensors, some obviously erroneous data caused by external factors such as human operation were excluded. The remaining data were used normally for plotting and were available for further analysis.

When testing the permeability coefficient of the soil, a minimum of three replicate soil specimens for permeability testing were collected from each substrate mix ratio to minimize measurement error. The permeability coefficients of the different XJTS substrate mixes before planting were determined using the falling head method. For the control group and the post-planting soil specimens, the constant head method was adopted for measurement, as preliminary tests indicated a substantial increase in their permeability coefficients.

## References

[1] Wang S., Song S., Zhang H., Yu L., Jiao C., Li C., Wu X., Zhao W., Best J., Roberts P., Fu B. (2025). Anthropogenic impacts on the Yellow River Basin. Nat. Rev. Earth Env..

[2] Xu Z., Zhang S., Yang X. (2021). Water and sediment yield response to extreme rainfall events in a complex large river basin: A case study of the Yellow River Basin, China. J. Hydrol..

[3] Benzerara M., Guihéneuf S., Belouettar R., Perrot A. (2021). Combined and synergic effect of algerian natural fibres and biopolymers on the reinforcement of extruded raw earth. Constr. Build. Mater..

[4] Zentar R., Wang H., Wang D. (2021). Comparative study of stabilization/solidification of dredged sediments with ordinary Portland cement and calcium sulfo-aluminate cement in the framework of valorization in road construction material. Constr. Build. Mater..

[5] Feng D., Liang B., Wan Y., Yi F., Liu L., Zhang Y., He X. (2025). Mechanical and water stability properties of biopolymer-treated silty sand. J. Rock Mech. Geotech. Eng..

[6] Feng D., Liang B., Sun W., He X., Yi F., Wan Y. (2023). Mechanical properties of solidified dredged soils considering the effects of compaction degree and residual moisture content. Dev. Built Environ..

[7] Feng D., Liang B., He X., Yi F., Xue J., Wan Y., Xue Q. (2023). Mechanical properties of dredged soil reinforced by xanthan gum and fibers. J. Rock Mech. Geotech. Eng..

[8] Gu J., Ni J., Liu S., Chen Y. (2025). Shear strength of biopolymer amended soil under freeze-thaw cycles: Experimental investigation and DEM modeling. Eng. Geol..

[9] Xu Y., Chena F. (2012). Effects of concrete content in vegetation concrete matrix on seed germination and seeding establishment of Cynodon dactylon. Procedia Eng..

[10] Zheng H., Cai M., Bai Y., Xu J., Xie Y., Song H., Li J., Gao J. (2021). The effect of guttation on the growth of bamboo shoots. Forests.

[11] Zhang J., Jia M., Jiang T., Kato S., Sun D., Gao Y., Yang Z. (2025). Dynamic deformation characteristics and microscopic analysis of xanthan gum-treated silty soil during wetting process. J. Rock Mech. Geotech. Eng..

[12] Feng D.Z., Zhang D.J., Jin J.X., Ran X.R., Liang B., Wan Y. (2026). Effect of biopolymer and plant fiber on soil-water characteristics of sandy soil. Sci. Rep..

[13] Fan T., Stewart B.A., Payne W.A., Yong W., Luo J., Gao Y. (2005). Long-term fertilizer and water availability effects on cereal yield and soil chemical properties in Northwest China. Soil Sci. Soc. Am. J..

[14] Gu J., Ni J., So P.S. (2026). Temperature-dependent soil water retention and hydraulic conductivity model for biopolymer-treated soil. Can. Geotech. J..

[15] Gu J., Ni J., Xu G., Zhou Y., Zhang H. (2026). Experimental and DEM investigation of thermal effects on mechanical properties of biopolymer treated soil. Transp. Geotechn..

[16] Chang I., Prasidhi A.K., Im J., Shin H.D., Cho G.C. (2015). Soil treatment using microbial biopolymers for anti-desertification purposes. Geoderma.

[17] Tran A.T.P., Chang I., Cho G.C. (2019). Soil water retention and vegetation survivability improvement using microbial biopolymers in drylands. Geomech. Eng..

[18] Pu S., Hou Y., Ma J., Zou Y., Xu L., Shi Q., Qian S., Pei X. (2019). Stabilization behavior and performance of loess using a novel biomass-based polymeric soil stabilizer. Environ. Eng. Geosci..

[19] Gao X., Li T., Li X., Cao X., Cui Z. (2022). Preparation of a newly synthesized biopolymer binder and its application to reduce the erosion of tailings. J. Environ. Manage..

[20] Che W., Liu J., Hao S., Ren J., Song Z., Bu F. (2022). Application of colloid-sand coating treated by a hydrophilic polysaccharide biopolymer material for topsoil stability control. Geoderma.

[21] Sorze A., Valentini F., Dorigato A., Pegoretti A. (2023). Development of a xanthan gum based superabsorbent and water retaining composites for agricultural and forestry applications. Molecules.

[22] Zhang C., Xie X.R., Duan Q.S., Zhang Y.K., Li X.S., Li S.F., Xu X.Q., Chen Z.F. (2024). Effects of root characteristics on the shear characteristics of root-soilcomplex in wood fiber reconstructed red soil. Trans. Chin. Soc. Agric. Eng..

[23] Ko D., Kang J. (2020). Biopolymer-reinforced levee for breach development retardation and enhanced erosion control. Water.

[24] Li L.H., Fan C.B., Bai Y.X., Li W.T. (2023). Mechanical properties and plant⁃growing of rice straw reinforced soil. China Civ. Eng. J..

[25] Tong X.D., Ci X., Sun R.Y. (2025). Preliminary study on ecological sand fixation effects of biopolymers. Chin. J. Geotech. Eng..

[26] Ni J., Chen J., Liu S., Hao G., Geng X. (2022). Experimental study of the usage of combined biopolymer and plants in reinforcing the clayey soil exposed to acidic and alkaline contaminations. Appl. Sci..

[27] Ren T.J., Yuan L.M., Gao Y., Wang C.Y., Jia R.T., Xu Z.Z. (2023). Experimental study on soil fixation performance of three plant-based sand fixing agents and their effects on plant growth. J. Soil Water Conserv..

[28] Tao G.L., Zhou H.J., Xiao H.L., Zhou H.Y. (2025). Mechanical and vegetative properties and anti-erosion effect of a new ecological slope protection material. Rock Soil Mech..

[29] Zhang H., Zhu H., Wu Y., Xu P., Hong C., Liu Y., Li G., Hu X. (2025). Mechanical properties of surface soil in alpine meadow and its relationship with soil cracking in Qinghai Province, China. J. Arid Land.

[30] Yao Q.Y., Kumar R., Liu W.L., Garg A., Jiang M.J. (2025). Mechanism of water retention and gas flux in unsaturated soils amended with four different pyrolyzed plant based biomass. Soil Use Manage..

[31] Wang X., Wang K., Deng T., Wang F., Zhao Y., Li J., Huang Z., Wang J., Duan W. (2024). Contribution of soil matric suction on slope stability under different vegetation types. J. Soils Sediments.

[32] Ng C.W.W., Liao J.X., Bordoloi S. (2022). Relationship between matric suction and leaf indices of Scheffiera arboricola in biochar amended soil. Can. Geotech. J..

[33] Rana A., Kumar A., Azizi A., Osman A.S., Toll D.G. (2025). Assessment of an amended soil as a climate adaptive barrier: Element testing and physical modelling. Geomech. Energy Environ..

[34] Feng D., Wan Y., Jin J., He X., Liang B., Xue Q. (2025). The effects of wetting-drying and freezing-thawing cycles on mechanical properties of biopolymer-fiber treated soil. Dev. Built Environ..

[35] Wan Y., Hui X., He X., Xue J., Feng D., Chen Z., Li J., Liu L., Xue Q. (2022). Utilization of flue gas desulfurization gypsum to produce green binder for dredged soil solidification: Strength, durability, and planting performance. J. Clean. Prod..

[36] Jin X., Wang J., Liu X., Chang J., Li C., Lu G. (2025). Potassium fulvate alleviates salt-alkali stress and promotes comprehensive growth of oats in saline-alkali soils of the Qaidam Basin. Plants.

