# Supplementary material for: Evaluation of the vegetative properties of biopolymer- and plant fiber-solidified sandy soil
Source: iScience. 2026 Jul 22;29(8):116921. doi: 10.1016/j.isci.2026.116921 (PMC13426210; doi:10.1016/j.isci.2026.116921)
Supplement: Document S1. Figures S1–S3 [file mmc1.pdf]

iScience, Volume *29*

Supplemental information

Evaluation of the vegetative properties of biopolymer- and plant fiber-solidified sandy soil

Dianzhi Feng, Dejiang Zhang, Jiaxu Jin, Bing Liang, and Yong Wan

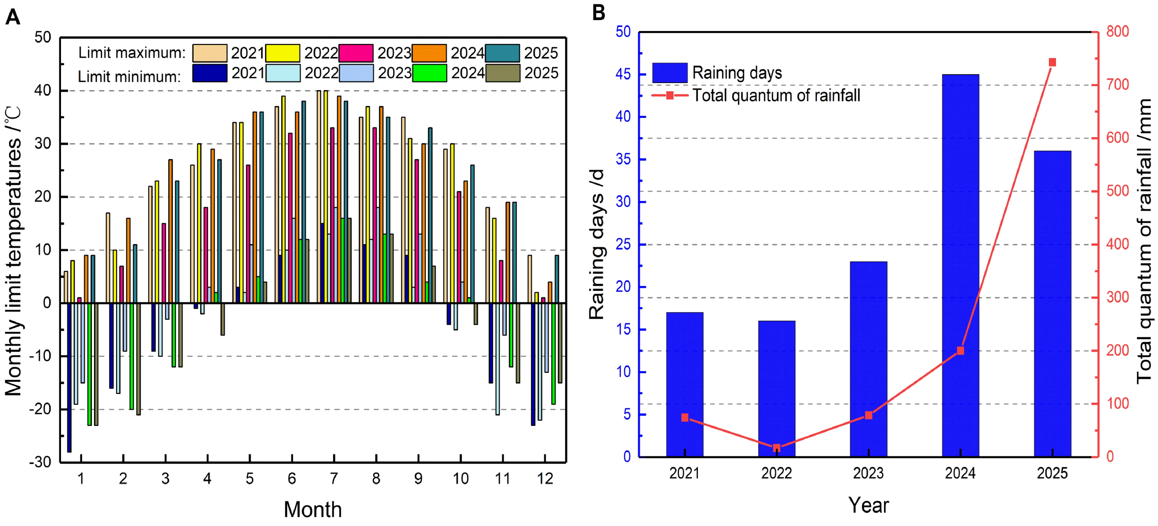

Figure S1. Temperature and precipitation changes from 2021 to 2025.

(A) Variations in monthly extreme temperatures.

(B) Annual raining days and total quantum of rainfall.

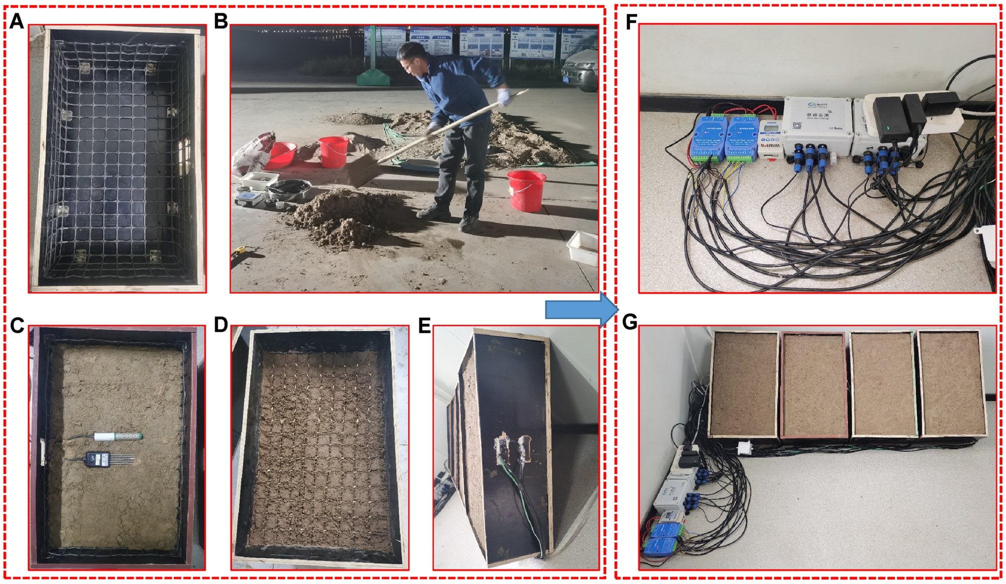

Figure S2. Flowchart of the indoor planting test in model boxes

(A) Planting model box.

(B) Mix the planting substrate.

(C) Pre-installed sensor.

(D) Sow plant seeds.

(E) Side view of planting model box.

(F) Sensor wiring and configuration.

(G) Plant growth monitoring.

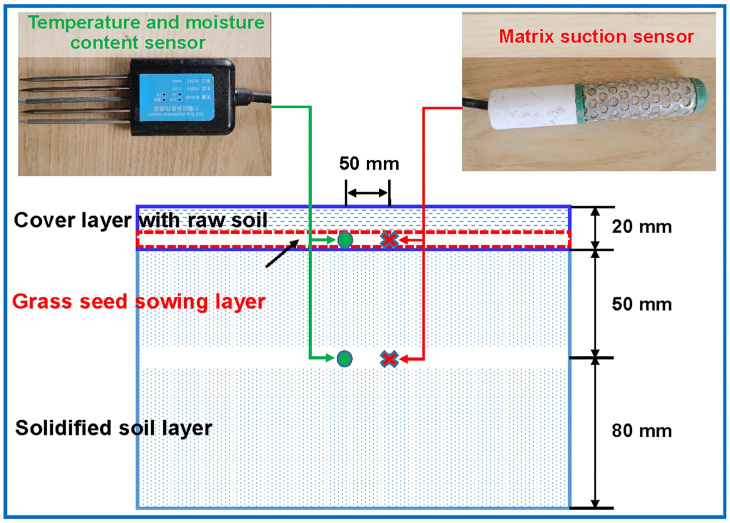

Figure S3. Illustration of sensor arrangement in the indoor planting test
